# Quantitative differences between common occupational health risk assessment models

**DOI:** 10.1002/1348-9585.12164

**Published:** 2020-09-19

**Authors:** Qiuliang Xu, Fang Yu, Fei Li, Hua Zhou, Kang Zheng, Meibian Zhang

**Affiliations:** ^1^ Zhejiang Provincial Center for Disease Control and Prevention Hangzhou Zhejiang China; ^2^ Zhejiang Zheng’an Testing Technology co. LTD Wenzhou Zhejiang China

**Keywords:** Methodology, Occupational health, Risk assessment, Workplace

## Abstract

**Objectives:**

Methodological studies on occupational health risk assessment (OHRA) models are rarely reported. This study aimed to explore the quantitative differences between common OHRA models.

**Methods:**

The risk ratios (RRs) in five typical industries (leather, wooden furniture manufacturing, printing and dyeing, printing, and garment manufacturing) were investigated using six OHRA models, namely the models from the US Environmental Protection Agency (EPA), Singapore, the Control of Substances Hazardous to Health (COSHH), Australia, Romania, and International Council on Mining and Metals (ICMM). The consistency, correlation, and reliability were evaluated for quantitative differences between the models.

**Results:**

The order of the RRs obtained from the EPA, Singaporean, and COSHH models in the five industries was consistent with the order of the inherent risk levels in those industries. The EPA and Singaporean models could effectively distinguish the inherent risk levels of risk factors like xylene and ethyl acetate. The order of RR between the six models was: RR _EPA_ > RR _COSHH_ > RR _Singaporean_ > RR _Australian_ > RR _Romanian_ and RR _ICMM_ (*P* < .05). The EPA model had the weakest correlations with other models. The Singaporean model had positive correlations in RRs with the other models (*P*＜0.01).

**Conclusions:**

The EPA and Singaporean models exhibited good reliability since they could distinguish the inherent risk of the industry or risk factor and tended to get higher risk levels. The EPA model was independent and the Singaporean model had a good correlation with other models. More studies on OHRA methodology are needed.

## 1 INTRODUCTION

Occupational health risk assessment (OHRA) is an effective tool to control the health risk of occupational hazardous factors in workplaces.^1^, ^2^ Many industrialized countries and international organizations have developed their own OHRA methods, including qualitative, quantitative, and semi‐quantitative ones. Currently, there are many common models used for OHRA, including the models from the United States Environmental Protection Agency (EPA),^3^ Singapore,^4^ the United Kingdom's Control of Substances Hazardous to Health Essentials (COSHH Essentials),^5^ Australia,^6^ Romania,^7^ and the International Council on Mining and Metals (ICMM).^8^ Based on the EPA, Singaporean, and COSHH models, China formulated a technical guideline for the OHRA of chemicals in the workplace (GBZ/T 298‐2017).^9^

Each model has its own advantages and limitations due to their different methodological principles. The EPA model, which is able to assess the carcinogenic and non‐carcinogenic risks of chemicals, has been widely used in many typical industries^10^, ^11^ because of its quantitative risk assessment based on epidemiological or toxicological data. However, the EPA model is limited to evaluating chemicals based on their reference concentrations (RfC) and inhalation unit risk (IUR). The Singaporean model, as a semi‐quantitative method, has been applied to evaluate the health risk in papermaking, chemical, electroplating, printing, and furniture manufacturing industries.^12^, ^13^ An exposure index method is an alternative when air monitoring data is absent; however, the model cannot assess physical factors, and its classification of exposure index is relatively rough. The COSHH model was reported to be applied in the lead‐acid battery manufacturing industry,^14^ and authors found it was simple and feasible, but might be prone to bias when judging liquid volatility. The Australian model was applied in battery manufacturing enterprises,^15^ and was found to have some shortcomings, such as relying on subjective judgment and professional knowledge; however, it was appropriate for OHRAs in small and medium‐sized enterprises. The Romanian model, which has some degree of subjectivity, was used in adhesives manufacturing enterprises^16^ and authors thought it was difficult to judge the probability of harmful consequences, but the calculation of the total risk level was an advantage.

At present, there are few reports on the methodological differences between OHRA models across industries. Different assessment results for the same hazard were often found when using different models,^11^, ^12^, ^13^, ^14^, ^15^, ^16^, ^17^, ^18^, ^19^ which largely depends on the methodological differences between OHRA models. A preliminary review by our team showed that quantitative, semi‐quantitative, and qualitative methods could be applied in combination when conducting OHRAs, since the scope and principles of these OHRA models are not exactly the same.^20^ Moreover we proposed a theoretical framework for comparing the qualitative and quantitative differences between different models^21^ and found that the strengths and limitations of OHRA models depended on their unique methodological principles and that combining the EPA, Singaporean, and COSHH models might be advantageous for developing an OHRA strategy.

China is one of the most occupational disease‐inductive countries in the world. Over 200 million workers from at least 20 million enterprises are at risk of occupational diseases in China.^22^ A total of nearly 1 million cases of occupational disease have been reported in China with nearly 30,000 reported cases per year.^23^ Developing countries are facing similar public health problems. OHRA can be used as a tool to control and manage the occupational health risks in these countries. Understanding the qualitative or quantitative differences of each model is fundamental for occupational health management in different industries. This study aimed to explore the quantitative differences between six common OHRA models (EPA, Singaporean, COSHH, Australian, Romanian, and ICMM) by evaluating five typical industries (leather, wooden furniture, printing and dyeing, printing, and garment). Thereby, we attempted to provide a basis for developing countries with a high prevalence of occupational disease to conduct methodological studies on OHRA and to strengthen occupational health risk management.

## 2 MATERIALS AND METHODS

### 2.1 Description of typical industries and factories

The leather, wooden furniture, printing and dyeing of cloth or textile, printing on paper, and garment manufacturing industries were selected as the typical industries for this study based on their inherent risks (IRs). The IRs of industries were directly obtained from a normative document formulated by a government department in China (namely, the “Management catalogue of occupational hazard risk classification for construction projects” issued by the State Administration of Work Safety of China).^24^ According to the document, each industry with occupational hazards is assigned a level of risk based on the advice and consultation of China's top occupational health experts. Therefore, in this study, the IR levels of the leather products and wooden furniture manufacturing industries were classified as “severe,” the printing and dyeing and printing industries were classified as "medium," and the garment manufacturing industry was classified as "low." Thus, the order of IRs between the five industries is: IR _Leather_ and IR _Furniture_ > IR _Printing and dyeing_ and IR _Printing_ > IR _Garment_.

A total of 50 enterprises in the five industries (10 enterprises per industry) from the Zhejiang province of East China were selected as typical factories. They comprised 1 large enterprise, 4 medium enterprises, 31 small enterprises, and 14 micro enterprises. Altogether 70% of them were small and medium‐ sized enterprises.^25^ Approximately 5,000 workers exposed to risk factors were involved.

### 2.2 Identification of risk factors

The risk factors and their exposure levels in the five industries are listed in Table 1. These factors were determined through field investigation, air sampling, and laboratory tests based on two occupational health standards in China, that is, the “Specifications of air sampling for hazardous substances monitoring in the workplace (GBZ 159)” and “Determination of toxic substances in workplace air (GBZ/T160 and 300).” The exposure levels of risk factors (eg n,n‐dimethyl formamide (DMF), wood dust, formaldehyde, xylene, butyl acetate, styrene, methyl acetate, toluene, and ethyl acetate) at various locations in the wooden furniture, leather, printing and dyeing, and printing industries had different degrees of exceeding the permissible concentration‐time weighted average (PC‐TWA) permitted by China or the American conference of governmental industrial hygienists(ACGIH) TWA permitted by the USA. This was not the case for the garment industry.

**Table 1:** General information and exposure levels of risk factors in five typical industries

Industry	Location	No. of locations	Risk factor	Exposure levels （Mean, range） （mg/m^3^）	Evaluation by China PC‐TWA	Evaluation by ACGIH TWA
Leather	Wet process ‐preparation	9	N,N‐Dimethyl formamide(DMF)	101（22.5‐586）	Disqualified	Disqualified
			Wood dust	5.4（0.9‐14.7）	Disqualified	Disqualified
	Wet process ‐placing	7	N,N‐Dimethyl formamide(DMF)	197.7（28.7‐753）	Disqualified	Disqualified
	Wet process ‐coating machine	9	N,N‐Dimethyl formamide(DMF)	68.2（8.7‐139）	Disqualified	Disqualified
	Dry process ‐preparation	10	N,N‐Dimethyl formamide(DMF)	59.9（7.9‐138）	Disqualified	Disqualified
			Methyl acetate	32.4（0.135‐186.6）	Qualified	Disqualified
	Dry process ‐placing	8	N,N‐Dimethyl formamide (DMF)	66.1（4.4‐206）	Disqualified	Disqualified
			Methyl acetate	34.2（0.135‐227.5）	Disqualified	Disqualified
	Dry process ‐coating machine	10	N,N‐Dimethyl formamide (DMF)	52.2（1.65‐230）	Disqualified	Disqualified
			Methyl acetate	66.3（0.135‐566.5）	Disqualified	Disqualified
	The third edition ‐preparation	7	N,N‐Dimethyl formamide (DMF)	10.9（1.65‐24.1）	Disqualified	Disqualified
			Methyl acetate	10.9（0.135‐44.1）	Qualified	Qualified
	The third edition ‐coating machine	10	N,N‐Dimethyl formamide (DMF)	56.1（5.3‐295）	Disqualified	Disqualified
			Methyl acetate	14.5（0.135‐114.4）	Qualified	Disqualified
Wooden furniture	Wood sawing	27	Wood dust	13.3（1.12‐33.3）	Disqualified	Disqualified
	Wood machining	102	Wood dust	17.08（0.7‐57.3）	Disqualified	Disqualified
	Manual processing of wood	28	Wood dust	11.3（2.4‐33.8）	Disqualified	Disqualified
	Clamping	32	Formaldehyde	0.206（0.034‐1.1）	Disqualified	Disqualified
	Polishing	40	Resin dust	15.8（2.3‐34.7）	Disqualified	/
	Paint modulating	10	Xylene	36.5（1.3‐348.2）	Disqualified	Disqualified
			Styrene	0.85	Qualified	Qualified
			Toluene‐2,6–diisocyanate（TDI）	0.0004（0.0001‐0.0006）	Qualified	Qualified
			Ethyl acetate	17.9（0.135‐138.5）	Qualified	Disqualified
			Butyl acetate	43.5（0.135‐392.4）	Disqualified	Disqualified
	Brushing paint	20	Xylene(all isomers)	25.2（1.65‐172.6）	Disqualified	Disqualified
			Styrene	0.85	Qualified	Qualified
			Toluene‐2,6–diisocyanate（TDI）	0.0016 (0.0001‐0.0065）	Qualified	Disqualified
			Ethyl acetate	6.8（0.135‐45.3）	Qualified	Qualified
			Butyl acetate	31.9（0.135‐241）	Disqualified	Disqualified
	Spraying paint	42	Xylene(all isomers)	24.55（0.165‐202.1）	Disqualified	Disqualified
			Styrene	12.9（0.85‐72.8）	Disqualified	Disqualified
			Toluene‐2,6–diisocyanate（TDI）	0.0006 (0.0001‐0.003）	Qualified	Disqualified
			Ethyl acetate	12.6（0.135‐100.7）	Qualified	Qualified
			Butyl acetate	27.9（0.135‐276.2）	Disqualified	Disqualified
Printing and dyeing	Dyeing	40	Hydrogen peroxide	0.4	Qualified	Qualified
			Acetic acid	0.1	Qualified	Qualified
	Clamping	19	Formaldehyde	0.29（0.034‐1.6）	Disqualified	Disqualified
	Modulating paint	14	Formaldehyde	0.23（0.034‐0.46）	Qualified	Disqualified
	Painting	11	Formaldehyde	0.21（0.034‐0.5）	Qualified	Disqualified
	Sewage treatment ‐station	10	Hydrogen sulfide	0.27	Qualified	Qualified
			Ammonia	0.42（0.065‐0.88）	Qualified	Qualified
Printing	Printing	10	Toluene	0.88（1‐74.3）	Disqualified	Disqualified
			Ethyl acetate	34.1（4.7‐78）	Qualified	Qualified
			Butyl acetate	48.3（1.1‐151）	Qualified	Disqualified
	Recombination	10	Toluene	1.24（1.2‐2.4）	Qualified	Qualified
			Ethyl acetate	54.1（0.9‐298.3）	Disqualified	Disqualified
			Butyl acetate	20.4（0.135‐52）	Qualified	Disqualified
Garment	Sewing	10	Fiber dust	0.39（0.1‐0.62）	Qualified	/
			Cotton dust	0.31（0.05‐0.5）	Qualified	Disqualified

Note

ACGIH TWA: American conference of governmental industrial hygienists time‐ weighted average concentration.

PC‐TWA: Permissible concentration‐time weighted average.

### 2.3 Methodology for ORHA modeling

The six common OHRA models (EPA, Singaporean, COSHH, Australian, Romanian, and ICMM) have similar assessment frameworks. All of them assess risk based on two factors: the inherent harmful consequences and their probability of occurrence, and they use four core steps, that is, hazard identification, hazard characterization, exposure assessment, and risk characterization. The detailed principles of the six models were previously reported in the literature.^3^, ^4^, ^5^, ^6^, ^7^, ^8^ Briefly, they were described as follows.

1. EPA model: The EPA inhalation risk assessment includes two components: carcinogenic and non‐carcinogenic risk assessments. In this study, only the non‐carcinogenic risk assessment was used.
  - Estimating exposure concentrations (EC):
    (1) $$\text{EC}=\left(\text{CA}\times \text{ET}\times \text{EF}\times \text{ED}\right)÷\text{AT}$$
    In this equation, EC (μg/m^3^) is the exposure concentration; CA (μg/m^3^) is the contaminant concentration in the air; ET (hours/day) is the exposure time; EF (days/year) is the exposure frequency; ED (year) is the exposure duration; and AT is the averaging time (ED [years] × 365 days/year × 24 h/day).
  - Non‐carcinogenic risk assessment:
    (2) HQ=EC/RfC
    In this equation, HQ is the hazard quotient and RfC represents the reference concentration for inhalation toxicity. The limit for HQ is considered to be 1.
2. The Singaporean model: The risk levels are calculated based on the hazard ratings (HR) and exposure ratings (ER), as shown in Equation 3:
  (3) $$\text{Risk}={\left(\text{HR}\times \text{ER}\right)}^{1/2}$$
  The HR is assigned based on the carcinogenicity classifications established by the International Agency for Research on Cancer (IARC). The ER is based on the ratio of the exposure level (E) and permissible exposure limit (PEL) or occupational exposure limit (OEL). If the exposure concentration is not available, exposure indices (EIs) can be used to determine the ER, as shown in Equation 4:
  (4) $$\text{ER}={\left[{\text{EI}}_{1}\times {\text{EI}}_{2}\times .\ldots {\text{EI}}_{n}\right]}^{1/n}$$
  EIs are determined using exposure factors or parameters of chemicals, such as vapor pressure, hazard control measures, the amount used per week, and duration of work per week.
3. The COSHH Essential model: This model simultaneously considers both the health hazards and exposure levels of chemical substances (solid or liquid), and uses a generic risk assessment to recommend the control level (one of the four types of approaches needed to achieve adequate control). The health hazard is determined based on allocation of the evaluated substance to a hazard band using a Risk‐phrase. The exposure potential is determined by allocating the substance to a dustiness or volatility band as appropriate, and another band is used for the scale of use.
4. The Australian model: The risk levels can be assessed using a manual diagram method or a calculator by analyzing the identified exposure levels, the possible consequences of exposure, and the likelihood of exposure for each hazard.
5. The Romanian model: Based on the severity of a hazard and probability of its occurrence, the concept of a risk acceptability curve was proposed. A matrix method is applied to qualitatively estimate the risk level.
6. The ICMM model: This model applies a matrix method to assess risk levels, including matrix combinations of health hazards and the probability of exposure occurring in a similar exposure group or process, as well as matrix combinations of health hazards and exposure levels with existing control measures.

### 2.4 **Risk ratio based on risk level conversion** ^21^

The risk levels obtained by the six OHRA models were different. The risk levels were converted to a risk ratio (RR) for quantitative comparisons between the models.

1. Risk level conversion: The risk level conversion is listed in Table 2. The risk assessment results of the EPA model were quantitative data, the risk assessment results of the COSHH model were the classification of control strategies, and the risk assessment results of the other four models were the classification of risk levels. In order to facilitate the comparison of the risk assessment results of each model, the EPA non‐carcinogenic risk assessment results, namely the hazard quotient (HQ), were converted into the risk level classification, which was divided into five levels, based on the classification standard of the Singaporean model. The risk assessment results of the COSHH model were also converted based on the Singaporean model.
2. Risk ratio: After the conversion, the assessment results of the six models were all converted into the classification of risk levels. The risk levels for the EPA, Singaporean, Australian and ICMM models were divided into five levels, the risk levels for the Romanian model was divided into seven levels, and the risk levels for the COSHH model were divided into 4 levels. In order to make the risk level of each method comparable, the concept of the RR was introduced, which was defined as the ratio between the risk level of a certain risk factor obtained from each model and the total risk level of the model. The RR could represent the relative risk level of risk factors obtained by one OHRA model, which made the risk levels obtained from different models comparable.

**Table 2:** Conversion of risk assessment results for the EPA and COSHH models

EPA model		COSHH model	
Hazard quotient (HQ)	Risk level^a^	Control strategy	Risk level^b^
＜0.1	1	‐	‐
0.1‐0.5	2	CS1	2
0.5‐1.0	3	CS2	3
1.0‐2.0	4	CS3	4
≥2.0	5	CS4	5

^a^ Modified based on the exposure rating method of the Singaporean model.

^b^ Modified based on the risk level of the Singaporean model.

### 2.5 Concentration ratio

In order to compare the exposure concentration of each risk factor in different locations, the concentration ratio (CR) was introduced, which was defined as the ratio between the exposure concentration of a risk factor and its corresponding OELs. The CR can represent the relative exposure level of a certain risk factor at a certain location.

### 2.6 Theoretical framework for quantitative comparison of six models

Quantitative comparisons were performed based on the analysis of RRs to test the reliability, consistency, and correlation of the models. The reliability of the model was verified by evaluating the consistency of an industry's RR obtained from each model with its own IR. In addition, the reliability of the model was also verified by comparing the consistency of a factor's RR obtained from each model with its own IR. In this study, xylene and ethyl acetate from the painting process in the wood furniture industry were selected as risk factors for evaluating the reliability of the model. The IR of a risk factor depends on its inherent hazardous consequences and exposure probability. The parameters in inherent hazardous consequence for xylene are as follows: the carcinogenesis classification from the International Agency for Research on Cancer (IARC) is G3, and the RfC value is 0.1mg/m^3^, while the carcinogenesis classification for ethyl acetate is not defined, and its RfC was 3.5mg/m^3^, which was calculated from the RfD based on a formula, that is, RfC = RfD×BW/DIR, where BW is the body weight (kg) and DIR is the daily expiratory volume (m^3^/d).^26^ It is clear that the inherent hazardous consequence of xylene is greater than that of ethyl acetate. The CR of xylene at the painting location [0.216(0.074‐0.518)] was greater than that of ethyl acetate [0.024(0.001‐0.053)] (*P* < .05). Therefore, the inherent risk of xylene at the painting location was higher than that of ethyl acetate. The consistence assessment was to analyze the statistical differences in RRs between the OHRA models. The correlations among OHRA models were evaluated based on the correlation coefficients.

### 2.7 Statistical analysis

The Kruskal‐Wallis H(K) method was used to analyze the RRs across different OHRA models or different industries. The Mann‐Whitney U method was used to analyze the RRs between xylene and ethyl acetate obtained from different OHRA models. The Spearman correlation analysis (abnormal distribution) was used to analyze the correlation of RRs.

## 3 RESULTS

### 3.1 The differences in reliability between the six OHRA models

**Figure 1:**
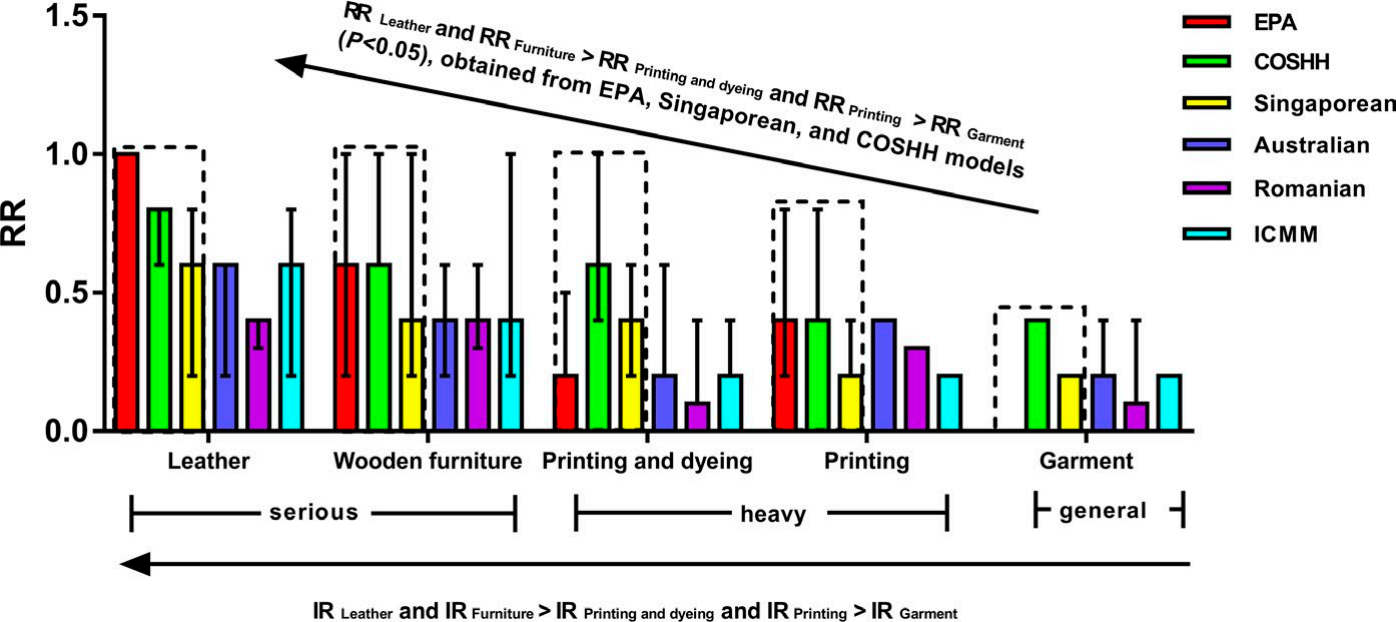
Quantitative comparisons of risk ratios (RRs) between the five industries using the six models. The EPA, Singaporean, and COSHH models could effectively distinguish the inherent risks (IRs) of the five industries using the RRs (*P* < .05)

Figure 1 and Table 3 show the results of the quantitative comparisons of RRs between the six OHRA models in the five industries. The order of RRs between the five industries obtained from the Singaporean model was: RR _Leather_ and RR _Furniture_ > RR _Printing and dyeing_ and RR _Printing_ > RR _Garment_ (*P* < .05), which was consistent with the order of IRs of the five industries (eg IR _Leather_ and IR _Furniture_ > IR _Printing and dyeing_ and IR _Printing_ > IR _Garment_). Similar results were observed from the EPA and COSHH models. The Australian, Romanian, and ICMM models could not distinguish the IR difference of industries using the RRs (*P* > .05).

**Table 3:** Quantitative comparisons in risk ratios between six models in five industries

Industry		Leather	Wooden furniture	Printing and dyeing	Printing	Garment	Sum
IR		Severe	Severe	Medium	Medium	Low	/
n		121	470	144	60	14	809
EPA	Risk level (range)	5	1‐5	1‐5	1‐5	/	1‐5
	RR [median (range)]	1.0^b^, ^c^, ^d^	0.6 (0.2‐1.0)^b^, ^c^	0.2 (0.2‐0.5)	0.4 (0.2‐0.8)	/	0.8 (0.2‐1.0) ^e^, ^f^, ^g^, ^h^, ^i^
COSHH	Risk level (range)	3‐5	2‐5	2‐5	2‐4	2	2‐5
	RR [median (range)]	0.8 (0.6‐0.8)^a^, ^b^, ^c^	0.6 (0.6‐1.0)^a^, ^b^, ^c^	0.6 (0.4‐1.0)^a^, ^b^	0.4 (0.4‐0.8)^a^	0.4	0.6 (0.6‐1.0)^e^, ^f^, ^g^, ^h^
Singaporean	Risk level (range)	1‐5	1‐5	1‐5	1‐3	1‐2	1‐5
	RR [median (range)]	0.6 (0.2‐0.8)^a^, ^b^, ^c^	0.4 (0.2‐1.0)^a^, ^b^, ^c^	0.4 (0.2‐0.6)^a^, ^b^	0.2 (0.2‐0.4)^a^	0.2 (0.2‐0.2)	0.4 (0.2‐0.8)^e^, ^f^, ^g^
Australian	Risk level (range)	1‐3	1‐3	1‐3	2	1‐2	1‐3
	RR [median (range)]	0.6 (0.2‐0.6)^b^, ^c^	0.4 (0.2‐0.6)^a^, ^b^, ^c^	0.2 (0.2‐0.6)^b^	0.4^a^	0.2 (0.2‐0.4)	0.4 (0.2‐0.6)^f^
Romanian	Risk level (range)	2‐3	2‐4	1‐3	2	1‐3	1‐4
	RR [median (range)]	0.4 (0.3‐0.4)^b^, ^c^, ^d^	0.4 (0.3‐0.6)^a^, ^b^, ^c^	0.1 (0.1‐0.4)^b^	0.3^a^	0.1 (0.1‐0.4)	0.3 (0.3‐0.4)
ICMM	Risk level (range)	1‐4	1‐5	1‐4	1‐4	1	1‐5
	RR [median (range)]	0.6 (0.2‐0.8)^a^, ^b^, ^c^, ^d^	0.4 (0.2‐1.0)^a^, ^b^, ^c^	0.2 (0.2‐0.4)^a^, ^b^	0.2 (0.2‐0.2)	0.2	0.2 (0.2‐0.8)

Note

IR: inherent risk according to the“Management catalogue of occupational hazard risk classification for construction projects”issued by the State Administration of Work Safety of China (2012 edition);

n: the number of risk level or risk ratio for all risk factors in each industry;

RR: risk ratio;

^a^ *P* < 0.05 compared with garment.

^b^ *P* < 0.05 compared with printing.

^c^ *P* < 0.05 compared with printing and dyeing.

^d^ *P* < 0.05 compared with wooden furniture.

^e^ *P* < 0.05 compared with ICMM model.

^f^ *P* < 0.05 compared with Romanian model.

^g^ *P* < 0.05 compared with Australian model.

^h^ *P* < 0.05 compared with Singaporean model.

^i^ *P* < 0.05 compared with COSHH model.

**Figure 2:**
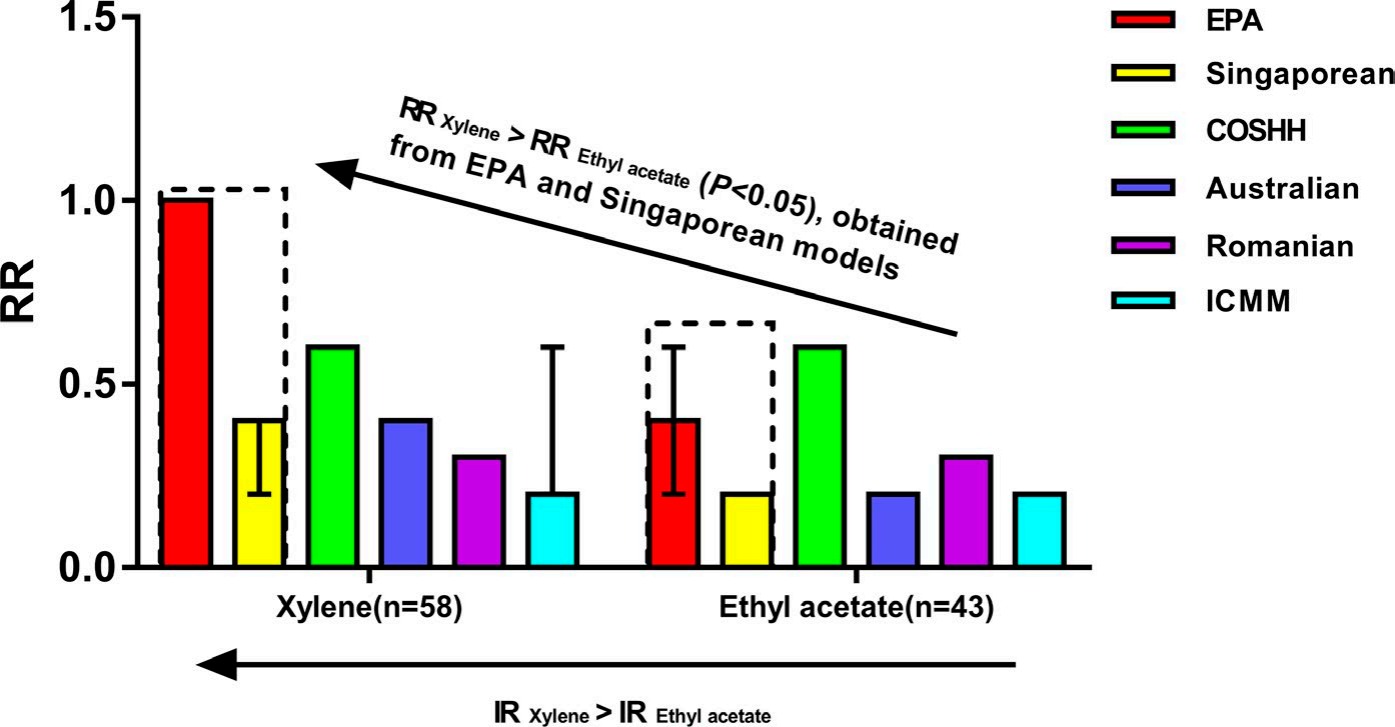
Quantitative comparison of risk ratios (RRs) between xylene and ethyl acetate at the painting location using the six models. The EPA and Singaporean models could effectively distinguish the inherent risks (IRs) of xylene and ethyl acetate using the RRs (*P* < .05)

Figure 2 shows the quantitative comparisons of RRs for xylene and ethyl acetate at the painting location using the six models. The RR of xylene obtained from the EPA and Singaporean models was significantly greater than that of ethyl acetate (*P* < .05), which was consistent with the difference in IR between the two chemicals (ie IR _xylene_ > IR _ethyl acetate_). Other four models failed to distinguish the IRs of the two chemicals using RRs.

### 3.2 The differences in consistency between the six OHRA models

Table 3 shows that the EPA model achieved the highest RR [0.8(0.2‐1.0)], respectively, followed by the COSHH model [0.6(0.6‐1.0)], the Singaporean model [0.4(0.2‐0.8)], the Australian model [0.4(0.2‐0.6)]. The Romanian model [0.3(0.3‐0.4)] and the ICMM model [0.2(0.2‐0.8)] had the lowest RR. The order of RRs among the six models was: RR _EPA_ > RR _COSHH_ > RR _Singaporean_ > RR _Australian_ > RR _Romanian_ and RR _ICMM_ (*P* < .05).

### 3.3 The correlation among the six OHRA models

Table 4 shows correlation analysis of RRs between the six models. The RR of the EPA model did not correlate with those of the COSHH, Romanian, and Australian models, and had a correlation with the ICMM model. The Singaporean model was positively correlated with the other five models (*P* < .01), and their correlation coefficients were relatively greater.

**Table 4:** Correlation analysis of risk ratios between the six models

	RR _EPA_	RR _Singaporean_	RR _COSHH_	RR _Australian_	RR _Romanian_	RR _ICMM_
RR _EPA_	1.000	‐	‐	‐	‐	‐
RR _Singaporean_	0.232*	1.000	‐	‐	‐	‐
RR _COSHH_	‐0.262	0.700*	1.000	‐	‐	‐
RR _Australian_	‐0.074	0.831*	0.652*	1.000	‐	‐
RR _Romanian_	‐0.014	0.819*	0.743*	0.874*	1.000	‐
RR _ICMM_	0.152*	0.887*	0.640*	0.857*	0.818*	1.000

* *P* < .01

## 4 DISCUSSION

In this study, the quantitative differences between common OHRA models were investigated regarding the three aspects of reliability, consistence, and correlation for five typical industries, using the RR.

The assessment results of reliability showed that the order of risk ratios for the five industries obtained by the EPA, Singaporean, and COSHH models, but not those obtained by the Romanian, Australian, and ICMM models, were consistent with each industry's own IR. This indicated that the EPA, Singaporean, and COSHH models were able to identify the occupational health risks more accurately than the other three models. This finding was supported by our preliminary study that reported that the risk ratios of the wood furniture manufacturing, electroplating, and crane manufacturing industries obtained by the EPA, Singaporean, and COSHH models were consistent with the inherent risk of these industries.^21^ The possible reasons for the reliability of the three models were that determining the inherent hazard level and the exposure level are relatively objective and accurate, in which determining the inherent hazard of risk factors is usually based on the data from animal experiments or epidemiological investigations, and the determination of exposure level is mainly based on the risk factor's physical and chemical properties, exposure concentration, or exposure time. However, the Australian, ICMM, and Romanian models are mainly based on the professional knowledge and working experience of the assessor when determining the hazard level and assessing the exposure level, which might lead to the subjectivity of the methodology and produce bias. Moreover according to a report on surveillance and occupational health risk assessment for key occupational diseases in Zhejiang province in the most recent ten years, which was provided by the Center for Disease Control and Prevention of Zhejiang province (Zhejiang CDC) of China, the leather industry and furniture manufacturing industry ranked 11th and 12th in risk level among 31 manufacturing sectors, followed by the printing and dyeing industry at 14th and the printing industry at 18th, and the garment industry at 26th. Therefore, the order of risk ratios of the five industries obtained by the EPA, Singaporean, and COSHH models was also consistent with the inherent risk of the five industries, which further confirmed the better reliability of the EPA, Singaporean, and COSHH models than the other models.

In this study, the inherent risk of xylene from the painting process was higher than that of ethyl acetate based on their inherent hazard and CRs. Inhalation of high concentrations of xylene can lead to coma or death in humans^27^, ^28^; low concentration exposure to xylene can cause occupational poisoning.^29^, ^30^ Ethyl acetate, as a low‐toxic chemical, mildly irritates the eyes and respiratory tract, even when it is inhaled in high concentrations, it may induce an anesthesia effect.^31^ The quantitative assessments of reliability between the two risk factors showed that the EPA and Singaporean models could effectively distinguish the difference in the IR level between xylene and ethyl acetate from the painting process, while other four qualitative OHRA models failed. This suggests that the COSHH model, as a qualitative OHRA method, was less reliable than the EPA and Singaporean models. The reason for the failure of the other four qualitative OHRA models to distinguish between the IR risk levels might be related to their weak ability in the exposure assessment of qualitative methodology. The exposure levels of chemicals evaluated by the COSHH model are based on the volatility and rough usage of chemicals. In this study, the volatility of xylene and ethyl acetate was similar and their amount of use in organic solvents used in the painting process could not be evaluated very accurately. In addition, the Australian, Romanian, and ICMM models determining the exposure levels of risk factors were greatly influenced by assessors’ subjective experience. In contrast, quantitative (eg the EPA model) or semi‐quantitative (eg the Singaporean model) methods adopt the real exposure concentration for exposure assessment.

The quantitative comparison of consistency showed that the order of risk ratios of the six models was RR _EPA_ > RR _COSHH_ > RR _Singaporean_ > RR _Australian_ > RR _Romanian_ and RR _ICMM_ (*P* < .05), which indicated that evaluating the same risk factor using different OHRA models would produce different risk levels. This finding was similar with the result observed by our research team in the previous study that the EPA, COSHH, and Singaporean models were prone to obtain higher risk ratios than the other three models in three industries (ie wooden furniture, electroplating, and crane manufacturing).^21^ Some scholars also found similar results.^32^, ^33^, ^34^, ^35^, ^36^ They found significant differences of risk levels between different OHRA models in typical industries such as the gas pipeline, electroplating, and chair furniture manufacturing industries. In the printing industry, a similar order of RRs between the six OHRA models was observed, that is, RR _EPA_ > RR _COSHH_ > RR _Singaporean_ and RR _Australian_ > RR _ICMM_ > RR _Romanian_ (*P* < .05).

The correlation analysis showed that the RR of the EPA model had the weakest correlation with other models and the RR of the Singaporean model was positively correlated with the other five models (*P*＜0.01), which suggested that the EPA model had an independence in methodology and the Singaporean model had a good correlation with the other models. The EPA model is based on quantitative data, which evaluates risk factors using its unique parameters, such as the IUR and RfC based on the epidemiological or toxicological data. The Singaporean model, as a semi‐quantitative method, based on both qualitative and quantitative data, possesses common characteristics of quantitative and qualitative models, and thus is able to make up for the shortcomings of the quantitative and qualitative methods, and generate a good correlation with other models. The other four models are qualitative methods based on qualitative data. This was in agreement with our preliminary reports that the EPA model was highly independent and had no correlation with the other five models and that the Singaporean model was related to all the models except the EPA model in three industries (wooden furniture, electroplating, and crane manufacturing).^21^

The main limitation of this study was the relatively small sample size of enterprises and industries. The study should be replicated in more industries and regions to observe if they perform similarly across multiple different samples.

## 5 CONCLUSIONS

The following conclusions can be drawn based on these findings: (a) the EPA and Singaporean models had higher reliability since they could distinguish the IR of the industry or risk factor, and tended to indicate higher risk levels; (b) the EPA model was relatively independent in methodology, and the Singaporean model had the strongest correlation with other models; (c) a combination of different methodologies could be a strategy for OHRAs.

More studies on the methodological differences of OHRA are needed with regard to the following aspects: (a) The theoretical framework of comparative studies between different models should be further improved; (b) the quantitative differences among models should be investigated in more industries in developing countries; (c) risk management strategies for different industries should be proposed based on risk assessment results for efficiently controlling the occupational hazard.

## DISCLOSURE

*Approval of the research protocol:* N/A;

*Informed Consent:* N/A;

*Registry and Registration Number of the study/trial:* N/A;

*Animal Studies:* N/A;

*Conflict of Interest:* None declared.

## Author Contributions

Qiuliang Xu collected and analyzed the data and wrote the manuscript; Meibian Zhang conceived the ideas and led the writing; Fang Yu, Fei Li, Hua Zhou, and Kang Zheng contributed to data collection and field investigation.

## References

[1] Gridelet L , Delbecq P , Hervé L , et al. Proposal of a new risk assessment method for the handling of powders and nanomaterials. Ind Health. 2015;53(1):56‐68. 25327299 10.2486/indhealth.2014-0046 PMC4331195

[2] Shur PZ , Zatseva NV , Alekseev VB , et al. Occupational health risk assessment and management in workers in improvement of national policy in occupational hygiene and safety. Gig Sanit. 2015;94(2):72‐75. 26155652

[3] USEPA . Risk assessment guidance for superfund volume I: human health valuation manual (Part F, supplemental guidance for inhalation risk assessment): EPA/540/‐R‐070‐/002[R]. Washington, DC: U.S. Environmental Protection Agency; 2009.

[4] Ministry of Manpower Occupational Safety and Health Division . A semi‐quantitative method to assess occupational exposure to harmful chemicals. https://www.wshc.sg/files/wshc/upload/cms/file/2014/A%20Semiquantitative%20Method%20to%20Assess%20Occupational%20Exposure%20to%20Harmful%20Che.pdf. Published 2014. Accessed April 10, 2020

[5] Health and Safety Executive . COSHH Essentials‐easy steps to control chemicals. https://www.researchgate.net/lite.publication.PublicationDownloadCitationModal.downloadCitation.html?fileType=RIS&citation=citation&publicationUid=31283880. Published 2000. Accessed April 10, 2020

[6] University of Queensland . Occupational health and safety risk assessment and management guideline. Brisbane: Occupational Health and Safety Unit; 2011.

[7] National Research Institute for Labour Protection . Risk assessment method for occupational accidents and diseases. http://www.protectiamuncii.ro/pdfs/risk_assessment_method.pdf. Published 1998. Accessed April 10, 2020

[8] International Council on Mining and Metals . Good practice guidance on occupational health risk assessment. Second edition. https://www.icmm.com/website/publications/pdfs/health‐and‐safety/161212_health‐and‐safety_health‐risk‐assessment_2nd‐edition.pdf. Published 2009. Accessed April 10, 2020

[9] National Health and Family Planning Commission . Guidelines for Occupational Health Risk Assessment of Chemicals in the Workplace: GBZ/T 298–2017. http://www.nhc.gov.cn/ewebeditor/uploadfile/2017/11/20171114161459715.zip. Published September 30, 2017. Accessed April 10, 2020.

[10] Leng P , Li X , Wang Q , et al. Occupational health risk assessment for workers exposed to low concentration of benzene. Environ Occup Med. 2018;35(11):985‐989. (in Chinese).

[11] Yuan W , Leng P , Zhou L , et al. Comparative study on occupational risk assessment using two foreign models. Environ Occup Med. 2015;32(1):51‐55. (in Chinese).

[12] Zhou L , Zhang M , Zou H , et al. Application of two health risk assessment models in the occupational health risk assessment of chemicals in different industries. PrevMed 2017;29(12):1217‐1222. (in Chinese).

[13] Xie H , Zhang M , Zhou L , et al. Application of two risk assessment models to the printing industry. Environ Occup Med 2016;33(1):29‐33. (in Chinese).

[14] Chen L , Qian XR , Zhao D , et al. Occupational hazards in a lead‐acid battery enterprise: a comparison study of three health risk assessment methods. Chin J Public Health. 2018;6:849‐853. (in Chinese).

[15] Wang SS , Zhang MB , Jiang GQ , et al. An application study of Australian occupational risk assessment model in a certain battery production enterprise. Zhejiang J Prev Med 2013;12:8‐11,48. (in Chinese).

[16] Zou Y , Lu L , Tang X , et al. Comparison of qualitative and semi‐quantitative occupational health risk assessment methods in an adhesive manufacturer. Chin Occup Med 2018;6:770‐774,778. (in Chinese).

[17] Bian GL , Wang AH , Li XH , et al. A comparative study on the application of different methods of occupation health risk assessment in small furniture manufacturing industry. Zhejiang J Prev Med 2017;29(10):1003‐1008. (in Chinese).

[18] Li XD , Ding J , Liu M ,, et al. Application research of three risk assessment methods to organic solvents in painting produced industry. Prev Med. 2018;30(8):794‐798. (in Chinese).

[19] Tian YF , Liu KQ , Wu LK , et al. Application of three models for occupational health risk assessment to a transformer factory in Shenzhen City. Occup and Health. 2018;34(18):2449‐2452. (in Chinese).

[20] Zhou LF , Tian F , Zou H , et al. Research progress in occupational health risk assessment methods in China. Biomed Environ Sci. 2017;30(8):616‐622. 28807103 10.3967/bes2017.082

[21] Tian F , Zhang M , Zhou L , et al. Qualitative and quantitative differences between common occupational health risk assessment models in typical industries. J Occup Health. 2018;60(5):337‐347. 29877200 10.1539/joh.2018-0039-OA PMC6176034

[22] Zhang ZB , Chen G , Zhang YY , et al. Explore to status, problem and measures against occupational hazards. J Saf Sci Technol 2014;S1:51‐54. (in Chinese).

[23] General Office of the State Council . (2017). Notice of the general office of the state council on the issuance of the national occupational disease prevention plan (2016–2020): Guo ban fa [2016] no. 100. http://www.gov.cn/zhengce/content/2017‐01/04/content5156356.htm. Published April 2017.

[24] State Administration of Work Safety . Management catalogue of occupational hazard risk classification of construction projects (2012 edition). https://www.mem.gov.cn/gk/gwgg/gfxwj/2012/201206/t20120604_242994.shtml. Published May 31, 2012. Accessed April 12, 2020

[25] National Bureau of Statistics . (2020). Notice of the National Bureau of Statistics on the issuance of measures on the classification of large, medium, small and micro enterprises by the National Bureau of Statistics. http://www.stats.gov.cn/tjgz/tzgb/201801/t20180103_1569254.html. Published December 2017. Accessed April 12, 2020

[26] Ministry of Environmental Protection . Exposure Factors Handbook of Chinese Population. Beijing: China Environment Press; 2013. (in Chinese).

[27] Sharada TR , Malathi N . Health hazards of xylene: a literature review. J Clin Diagn Res. 2014;8(2):271‐274. 10.7860/JCDR/2014/7544.4079 PMC3972585 24701554

[28] Kandyala R , Raghavendra SC , Xylene RST . An overview of its health hazards and preventive measures. J Oral Maxillofac Pathol. 2010;14(1):1‐5. 21180450 10.4103/0973-029X.64299 PMC2996004

[29] Niaz K , Bahadar H , Maqbool F , et al. A review of environmental and occupational exposure to xylene and its health concerns. EXCLI J. 2015;14:1167‐1186. 26862322 10.17179/excli2015-623 PMC4743476

[30] Mohammadyan M , Baharfar Y . Control of workers’ exposure to xylene in a pesticide production factory. Int J Occup Environ Health. 2015;21(2):121‐126. 25487643 10.1179/2049396714Y.0000000098 PMC4457120

[31] International Labor Organization . ETHYL ACETATE. https://www.ilo.org/dyn/icsc/showcard.display?p_lang=en&p_card_id=0367&p_version=2. Published April 2014.

[32] Xu QL , Zhang MB , Zou H , et al. Quantitative comparison of six common occupational health risk assessment models for small printing companies. J Environ Occup Med 2020;37(2):131‐137. (in Chinese).

[33] Li‐Fang Z , Mei‐Bian Z . Research progress on occupational health risk assessment methodology. J Environ Occup Med 2020;37(2):125‐130. (in Chinese).

[34] Xi XJ , Li T , Huang L . Application of two types of occupational health risk assessment methods used in gas pipeline engineering. Environ Prot Oil Gas Fields 2017;27(3):55‐59. (in Chinese).

[35] Yuan WM , Fu H , Zhang MB , et al. The application of five foreign occupational hazard risk assessment models in a certain electroplating enterprise. Chin J Ind Hyg Occup Dis 2014;32(12):965‐967. (in Chinese).

[36] Zhang P , Liu T , Li H , et al. Applied study and case report of comparison and application of two occupational health risk assessment methods in chair furniture manufacturing enterprises. Prev Med 2018;30(2):158‐162. (in Chinese).

